# Detection of First Marker Trait Associations for Resistance Against *Sclerotinia sclerotiorum* in *Brassica juncea*–*Erucastrum cardaminoides* Introgression Lines

**DOI:** 10.3389/fpls.2019.01015

**Published:** 2019-08-06

**Authors:** Kusum Rana, Chhaya Atri, Javed Akhatar, Rimaljeet Kaur, Anna Goyal, Mohini Prabha Singh, Nitin Kumar, Anju Sharma, Prabhjodh S. Sandhu, Gurpreet Kaur, Martin J. Barbetti, Surinder S. Banga

**Affiliations:** ^1^School of Agricultural Biotechnology, Punjab Agricultural University, Ludhiana, India; ^2^Department of Plant Breeding and Genetics, Punjab Agricultural University, Ludhiana, India; ^3^School of Agriculture and Environment and the UWA Institute of Agriculture, The University of Western Australia, Crawley, WA, Australia

**Keywords:** Indian mustard, alien introgression, *Erucastrum cardaminoides*, genotyping by sequencing, quantitative trait loci, Genomic *in situ* hybridization

## Abstract

A set of 96 *Brassica juncea*–*Erucastrum cardaminoides* introgression lines (ILs) were developed with genomic regions associated with *Sclerotinia* stem rot (*Sclerotinia sclerotiorum*) resistance from a wild *Brassicaceous* species *E. cardaminoides*. ILs were assessed for their resistance responses to stem inoculation with *S. sclerotiorum*, over three crop seasons (season I, 2011/2012; II, 2014/2015; III, 2016–2017). Initially, ILs were genotyped with transferable SSR markers and subsequently through genotyping by sequencing. SSR based association mapping identified six marker loci associated to resistance in both A and B genomes. Subsequent genome-wide association analysis (GWAS) of 84 ILs recognized a large number of SNPs associated to resistance, in chromosomes A03, A06, and B03. Chromosomes A03 and A06 harbored the maximum number of resistance related SNPs. Annotation of linked genomic regions highlighted an array of resistance mechanisms in terms of signal transduction pathways, hypersensitive responses and production of anti-fungal proteins and metabolites. Of major importance was the clustering of SNPs, encoding multiple resistance genes on small regions spanning approximately 885 kb region on chromosome A03 and 74 kb on B03. Five SNPs on chromosome A03 (6,390,210-381) were associated with LRR-RLK (receptor like kinases) genes that encode LRR-protein kinase family proteins. Genetic factors associated with pathogen-associated molecular patterns (PAMPs) and effector-triggered immunity (ETI) were predicted on chromosome A03, exhibiting 11 SNPs (6,274,763-994). These belonged to three R-Genes encoding TIR-NBS-LRR proteins. Marker trait associations (MTAs) identified will facilitate marker assisted introgression of these critical resistances, into new cultivars of *B. juncea* initially and, subsequently, into other crop *Brassica* species.

## Introduction

*Brassica juncea* (2*n* = 36; AABB) or Indian mustard, is a premier oilseed crop of India, contributing nearly 28% of total edible oil supplies (Kumar, 2012). It is also a crop of considerable significance in China (Li et al., 2009; Yang et al., 2016), Canada (Woods et al., 1991) and more recently in Australia (Oram et al., 2005). It is an allotetraploid that arose through multiple independent hybridization events between wild forms of *Brassica rapa* and *Brassica nigra* (Burkill, 1930; Olsson, 1960; Vaughan et al., 1963; Axelsson et al., 2000; Prakash et al., 2009; Kaur et al., 2014). Traditional breeding approaches have helped to enhance crop productivity by exploiting within the species variation. However, these approaches have failed to address inherent susceptibilities of the species to the major biotic stresses. Absence of genetically characterized sources of resistance in the primary gene pool has been a major bottleneck. Of the diseases occurring on mustard, *Sclerotinia sclerotiorum*, the causal agent of *Sclerotinia* stem rot, is particularly damaging (Purdy, 1979; Bolton et al., 2006; Saharan and Mehta, 2008). It causes extensive yield losses world-wide including India (Shivpuri et al., 2000; Ghasolia et al., 2004), Australia (Kirkegaard et al., 2006), Germany (Horning, 1983), Canada (Morrall et al., 1976), United Kingdom (Hims, 1979; Rawlinson and Muthyala, 1979), and China (Li et al., 1999). While available cultural and chemical controls can reduce the severity of *Sclerotinia* stem rot, management practices are often inconsistent and do not provide effective and reliable control of *Sclerotinia* stem rot (Barbetti et al., 2014, 2015). Host resistance offers the only economic and sustainable method for managing this disease.

Partial resistance against this pathogen has been observed in certain germplasm lines of sunflower (Godoy et al., 2005), beans (Gilmore et al., 2002), peas (Porter et al., 2009), peanut (Cruickshank et al., 2002), and soybean (Hartman et al., 2000). Incomplete resistance was also identified in some *Brassica napus* and, to a lesser extent in *B. juncea*, genotypes from China (Li et al., 1999, 2006, 2009; Zhao et al., 2004; You et al., 2016), Australia (Li et al., 2006, 2009; You et al., 2016), and India (Singh et al., 2008, 2010; Goyal et al., 2011; Uloth et al., 2013; You et al., 2016). Resistance was generally quantitative and intermediate in its expression. Genetic investigations involving bi-parental populations and germplasm assemblages have helped to identify minor quantitative trait loci (QTL) with small effects, but these explained only a fraction of available variation. Significant involvement of homoeologous duplicated regions in the genetic control of quantitative resistance against *Sclerotinia* stem rot has been reported in *B. napus* (Fomeju et al., 2014; Gyawali et al., 2016; Behla et al., 2017). Other studies included attempts to understand defensive responses of the host; for example, those of *B. napus* to *Sclerotinia* infection by performing transcriptome (Zhao et al., 2009; Wei et al., 2016) or microarray (Zhao et al., 2007) analysis. Transcriptomics suggested activation of the plant immune system, and a possible role for sulfur metabolism and/or glucosinolates in the response to *S. sclerotiorum* attack. Genes specific to the resistant genotype, especially those relating to one or more specific defense responses (Anderson et al., 2018), the jasmonic acid pathway, lignin biosynthesis, signal transduction or encoding transcription factors, are known to be up-regulated (Zhao et al., 2007; Wei et al., 2016).

Identifying sources of resistance in *Brassica* is challenging as there is considerable variation in plant host responses from even small changes in environmental conditions, from using different disease screening techniques, and also as a result of the variation in aggressiveness across *Sclerotinia sclerotiorum* isolates (Li et al., 2007; Ge et al., 2012). There is a broad convergence of opinion for the need to enhance the level of genetic diversity present within *Brassica* crops to manage this pathogen (Barbetti et al., 2011, 2012; Uloth et al., 2013). Therefore, attempts have been made by our group to exploit alternate alleles from the related species in the family *Brassicaceae* (Banga and Banga, 2016). For the current studies, we selected a wild species, *Erucastrum cardaminoides*, that grows wild under the conditions of environmental stress in the Micronesian region (Warwick et al., 2000). Its history of evolution makes it a likely source of gene(s) against many biotic stresses, including *Sclerotinia* stem rot. In addition, this species is genetically close to *Brassica* (Gomez-Campo et al., 1999) as was confirmed by the cytogenetic analysis of intergeneric hybrids between *E. cardaminoides* with *B. rapa* and with *B. nigra* (Chandra et al., 2004). The synthetic alloploids between *E. cardaminoides* and *B. rapa* also showed higher degree of resistance to many biotic stresses, when compared with mustard checks. This encouraged us to transfer *E. cardaminoides* resistances to *B. juncea* by developing *B. juncea*–*E. cardaminoides* introgression lines (ILs) (Chandra et al., 2004; Garg et al., 2010). To facilitate that, we first developed a synthetic allotetraploid (2*n* = 38; AAEE) by hybridizing E. *cardaminoides* and *B. rapa*, followed by chromosome doubling. Strategy of using synthetic allotetraploid as a bridging species was followed as we had previously failed to produce a fertile hybrid between *E. cardaminoides* (2*n* = 18; EE) and *B. juncea* (2*n* = 36; AABB). The allotetraploid (2*n* = 38; AAEE) could be hybridized with *B. juncea* (2*n* = 36; AABB) to develop a F_1_ hybrid (2*n* = 37; AABE). Resultant plants were partially fertile and could be backcrossed with *B. juncea* to produce BC_1_ progeny, with {AAB (8)B(1–8) E (1–9)} as the likely chromosome configuration. Partially fertile BC_1_ plants were then bud pollinated to generate over 350 BC_1_S_1_ plants. These plants varied for pollen grain fertilities (45–89%) and chromosome numbers. Genome size analysis through flow cytometry and meiotic analysis of BC_1_S_1_ plants allowed us to identify segregants with apparent genome size equivalence with B. *juncea* and meiotic configuration of 18II + 1-4I. Of these, 18II may suggest a complete recovery of *B. juncea* with addition of 1–4 univalent from *E. cardaminoides*. Such a scenario is theoretically possible because chromosomes in monosomic dose are known to move asymmetrically toward opposite poles during meiotic anaphase. In extreme cases, all the univalent of a genome can end up at the same pole. Production of unreduced gametes is fairly common in *Brassica* aneuploids. Over 150 BC_1_S_1_ plants were then selfed, following single seed descent method. Three cycles of selfing, selection for high pollen grain fertility and cytogenetic analysis helped us to select about 100 plants with euploid chromosome number (2*n* = 36) in BC_1_S_4_ generation. We avoided second cycle of backcrossing with *B. juncea* to prevent expedited elimination of E genome chromosomes. Retention of such monosomic addition chromosomes over more cycles of selfing may help to improve chances of recombination between crop (A/B) and wild (E) genomes. Strict selection regime followed resulted in the availability of a relatively smaller number (<100) of stable *B. juncea*–*E. cardaminoides* ILs with higher fertility and euploid chromosome number for genotyping in BC_1_S_6_. Smaller sample size of ILs is always a limitation in wide hybridization programs.

Although association mapping is now considered a method of choice to resolve quantitative variation (Risch and Merikangas, 1996; Nordborg and Tavare, 2002), it is rarely used to understand the introgressed variation as attempted in the current study. ILs, in the present context, are fully fertile with euploid chromosome number and have stabilized in form of translocation homozygotes. Size of introgressed segment was possibly the defining factor in ILs and physical linkage strongly influenced linkage disequilibrium (LD) between molecular marker(s) and causative polymorphisms. This provided the genetic basis for association mapping of genes underlying resistance responses. The extent of LD of linked markers in the entire ILs set, was significantly higher than that of unlinked markers. This manuscript documents outcomes from screening *B. juncea*–*E. cardaminoides* ILs against a virulent isolate of *S. sclerotiorum* and subsequent genome wide association mapping to identify the genomic regions that are responsible for this resistance. Identified marker candidates will allow rapid introgression of these critical resistances, into new cultivars of *B. juncea* initially and, subsequently, into the many other crop and horticultural *Brassica* species.

## Materials and Methods

### Plant Materials

A total of 96 *B. juncea*–*E. cardaminoides* ILs were developed previously through wide hybridization between *B. juncea* and *E. cardaminoides* as described earlier.

### Chromosome Preparation and *in situ* Hybridization

We followed the method for preparation of chromosome spreads as described in Rana et al. (2017). To prepare species specific genomic *in situ* hybridization (GISH) probes, purified DNA of *B. cardaminoides* and, *B. nigra* and *B. juncea* were extracted using the DNeasy plant mini kit (Qiagen) as per manufacturer’s specifications. Genomic DNA of *E. cardaminoides* was labeled with rhodamine-5-dUTP dye (red color). DNA of *B. nigra* was labeled with fluorescein-12-dUTP dye (green) using a nick translation kit (Roche, Germany). We followed a two-step hybridization to perform GISH as described earlier (Rana et al., 2017). Visualization was carried out with Zeiss fluorescent microscope (ImagerZ2 AX10). Digital images were captured using Isis^®^ software. Images were cropped and optimized with Image J using only functions affecting the whole image.

### Screening of ILs for Resistance Responses to *S. sclerotiorum* Inoculation

For field evaluations, ILs were planted in a randomized complete block design, with 20 plants in paired rows of 2 m length with inter-row spacing 30 cm. The phenotypic screening was carried out in two replications. Foggers (with one fogger per 9 m^2^) were used to maintain high humidity for disease development, operating 2–3 times a day for 15 min each. Phenotyping of ILs for their resistance responses was carried out during 2011–2012 (Season I), 2014–1015 (Season II), and 2016–2017 (Season III). Stem inoculations were carried out by using isolate PAU-4, an aggressive and virulent strain of *S. sclerotiorum* collected from infested *Brassica* fields at Punjab Agricultural University, Ludhiana, India. PAU-4 is a known highly virulent isolate on *B. juncea* (Rana et al., 2017). A sclerotium was first surface sterilized, cultured and sub-cultured using standard procedures (Clarkson et al., 2003; Li et al., 2006). The parents, viz. the recipient (*B. juncea* cv. RLC-1) as well as the donor (*E. cardaminoides*), were used as susceptible and resistant checks, respectively. The stem inoculation method (Buchwaldt et al., 2005; Bradley et al., 2006) was used for disease inoculation. The ILs was evaluated for disease incidence and development at 4–5 weeks post-inoculation. Ten plants per genotype per replication were scored for disease incidence by measuring stem lesion lengths according to the method described by Li et al. (2006, 2007). The resistance responses of ILs were categorized into five classes, based on stem lesion length viz., highly resistant (HR); (0 < 2.5 cm), resistant (R); (2.5 < 5.0 cm), moderately resistant (MR); (5.0 < 7.5 cm), susceptible (S); (7.5 < 10.0 cm), and highly susceptible (HS); (>10.0 cm) as categorized earlier by Garg et al. (2010). Analysis of variance (ANOVA) was undertaken and standard deviation and standard errors estimated using SAS software (SAS Institute Inc. [SAS], 2013).

### DNA Extraction

A total of 96 ILs comprising all five disease expression categories (HR, R, MR, S and HS) were used for molecular profiling. DNA was harvested from young leaves with minor modifications of CTAB (Cetyltrimethylammonium Bromide) standard procedure (Doyle and Doyle, 1990). Isolated DNA was further digested with RNase at 37°C for 1 h. DNA was quantified by NanoDrop^®^ 2000 spectrophotometer (Thermo Scientific^TM^, Wilmington, DE, United States) and for purity by assessing the OD260/OD280 ratio and Qubit^®^ 2.0 flurometer (Thermo Scientific^TM^, Wilmington, DE, United States) quantitation to measure DNA concentration. DNA samples with OD260/OD280 ratio of 1.8–2.0 and total amount >1.5 ug were qualified for library construction.

### SSR Genotyping

A total of 96 ILs, along with resistant and susceptible parents, were first genotyped by using 100 polymorphic SSR markers. These markers were identified as transferable based on studies involving over 650 A and B genome specific markers (Kim et al., 2009; Lowe et al., 2004). Sequences of 48 B-genome specific SSRs were obtained under MTA from Isobel Parkin (Agriculture and Agri-Food, Canada). PCR reactions were performed in a 384-well Applied Biosystems thermocycler (Model EN61328). Amplified DNA product was fractioned using an automated high-throughput electrophoresis system (Caliper Lab Chip GX version 3.0.618.0, Caliper Life Science, United States). Allelic polymorphism of all markers was recorded and mapping positions were inferred from published data (Lowe et al., 2002, 2004; Kim et al., 2009).

### Genotyping by Sequencing

A smaller set of 84 ILs was genotyped by sequencing (GBS) (Elshire et al., 2011). Genomic services were outsourced (Novogene, Hong Kong). For this, high quality DNA of each sample was digested with appropriate combination of restriction enzymes based on *in silico* evaluation. This step was followed by several rounds of PCR amplification. Samples were then individually pooled and size-selected for the required fragments to complete the library construction. High quality libraries with appropriate insert sizes were then used for pair-end sequencing on Illumina^®^ HiSeq platform, with the read length of 150 bp at each end. The sequences and corresponding sequencing quality information were stored in a FASTQ file. The adapter sequences were removed from raw reads using the software Cutadapt (Martin, 2011). The available genome sequence of *B. juncea* v1.5^1^ (Yang et al., 2016) was used for reference based alignments of whole genome sequences (25×) from the four most prominent ILs (>25×), using software bowtie2 (Langmead and Salzberg, 2012). Initially, one introgression line was aligned into the reference genome and SNP called using the NGSEP-GBS pipeline (Duitama et al., 2014). Total SNPs were replaced in genome reference using a perl script, pseudomaker.pl implemented in SEG-Map (Zhao et al., 2010) to construct the first step mock-up pseudomolecules, which were then used as a reference for next ILs. This process was repeated four times to construct final mock-up reference for alignment of sequence tags. Identification of SNPs was carried out by using NGSEP-GBS pipeline (Duitama et al., 2014) after aligning the paired end reads of 84 introgressed lines on final mock-up reference genome. The resulting marker dataset comprised 30,863,034 SNPs. These were then filtered to include only quality SNPs for further analysis. Filtering parameters were: minimum mapping quality (30), minor allele frequency (0.1), only bi-allelic SNPs, minimum number of samples genotyped (65), maximum observed heterozygosity (30) and maximum missing calls (30%) were used for finding putative SNPs. After filtering, 78,578 SNPs were identified and imputed using fcgene and Beagle (Browning and Browning, 2016) software.

### Association Mapping Based on SSR Genotyping

The normalization of phenotypic data was done by using PBTools software^2^ in R-Package version 1.5 (R Core Team, 2013). The marker trait associations (MTAs) were identified by using two models executed in TASSEL version 2.1 (Bradbury et al., 2007^3^). GLM (generalized linear model) and MLM (mixed linear model) models were used. Bayesian model-based software, STRUCTURE version 2.2 (Pritchard et al., 2000), was used to determine the population structure by using multi-locus (SSR) genotypic data. Resultant Q-matrix was used as a covariate during association mapping analysis to reduce the bias from population structure. Association mapping was implemented in TASSEL software version 2.1 (Bradbury et al., 2007), measuring the non-random association between marker alleles from different loci (Zhu et al., 2008). Squared correlation coefficients between marker-trait data (*R*^2^ values) and associated probabilities were calculated and converted into –log10(*P*) values. The associated values are calculated with a false discovery rate (FDR) of 0.09 (Storey and Tibshirani, 2003) to reduce the false marker-trait associations. LD, also known as gametic phase disequilibrium, was established between markers (Flint-Garcia et al., 2003). Annotation, or gene prediction study, of significant markers was carried out using MEGANTE software (Numa and Itoh, 2014)^4^.

### Genome-Wide Association Analysis (GWAS) Based on SNP Genotyping

For genome-wide association analysis (GWAS), resistance responses (in terms of lesion length) were first converted into rank data and then transformed to log(x) for three crop seasons. These were also pooled over seasons. A principal component analysis (PCA) was performed across the introgressed lines to identify population stratification by MVP-GWAS tool. We used the imputed dataset of 78,578 SNP markers to calculate the PCs. First three components showed maximum variance. MVP tool^5^ was used for marker trait association with two different models, MLM and Farm CPU. GWAS were performed using MLM association accounting for kinship, and GLM and Farm CPU were selected with PCs as covariate in MVP tool. R software package “adegenet”^6^ was used for applying discriminant analysis of principal components (DAPC) in the association analysis. After DAPC correction, first three discriminant function was used as covariate in GLM model and kinship for MLM model for association analysis by software TASSELv5.2 (Bradbury et al., 2007). Manhattan plots were generated with multi-model plotting using MVP tools.

### SNP Validation

Six peak SNPs associated with trait variation were identified for validation. Primers were designed from flanking region of SNP using Primer 3 software and their thermodynamic properties were confirmed by Vector NTI. We selected eight ILs that differed for resistance responses. Genomic DNA from these test genotypes were amplified using designed primers. The PCR products were then purified and used for Sanger sequencing.

### *In silico* Prediction of Candidate Genes

We used 25 kb flanking regions on each side of resistance associated peak SNP found to predict candidate genes using *B. juncea* pseudomolecules as a reference. The predicted genes and their orthologous sequences were then annotated by BLAST run against the *A. thaliana* database using Blast2GO v5.2.5 tool (Götz et al., 2008). *Arabidopsis* protein database was used for gene finding as well as blast search. Protein IDs generated allowed annotation against all flowering plant databases (NCBI). These were further enriched by the biological functions inferred from the putative *Arabidopsis* orthologs. We used the gene ontologies of *Arabidopsis* orthologs for all analysis because they are far better curated than for *B. juncea*. Positions of the predicted candidate genes w.r.t. the SNPs, were detected by blast searching sequences from the predicted genes against *B. juncea* mock-up pseudomolecules. Functions of the predicted candidate genes were verified from literature to determine their relevance for the trait in question.

## Results

**FIGURE 1:**
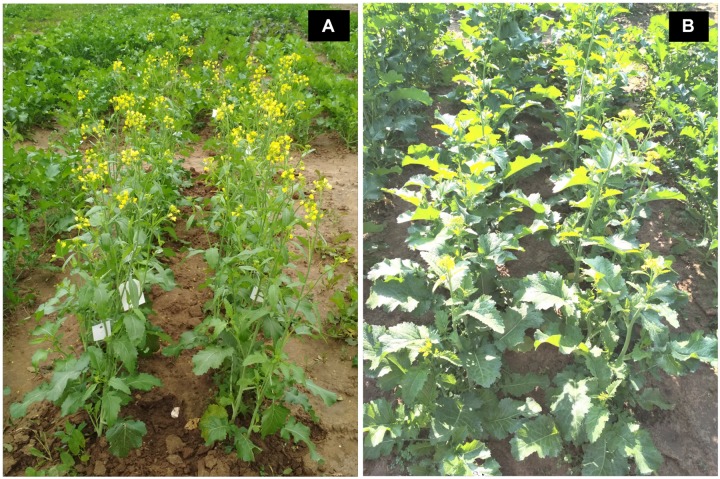
Field photographs showing plant morphology of **(A)** natural *Brassica juncea* as compared to **(B)** *B. juncea*–*E. cardaminoides* introgression line.

**FIGURE 2:**
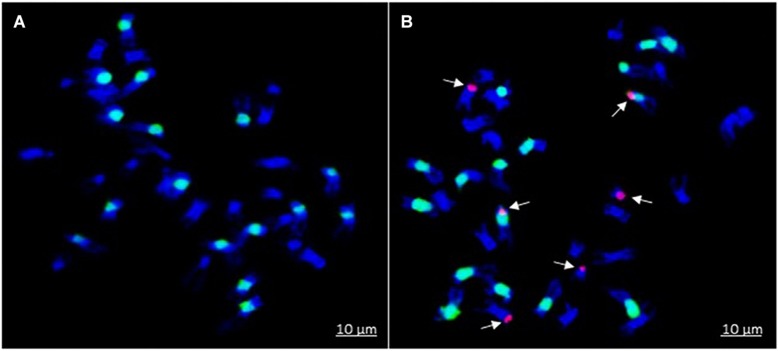
Genomic *in situ* hybridization on mitotic spreads of *B*. *juncea–E. cardaminoides* introgression lines (ILs). *B. nigra* B genome is painted in green (labeled with fluorescein-12-dUTP dye) while *E. cardaminoides* introgressions are shown in red color (labeled with rhodamine-5-dUTP dye): **(A)** *B*. *juncea* with no introgression; and **(B)** IL with segment substitutions in two chromosome pairs of A-genome and one chromosome pair of B genome.

**FIGURE 3:**
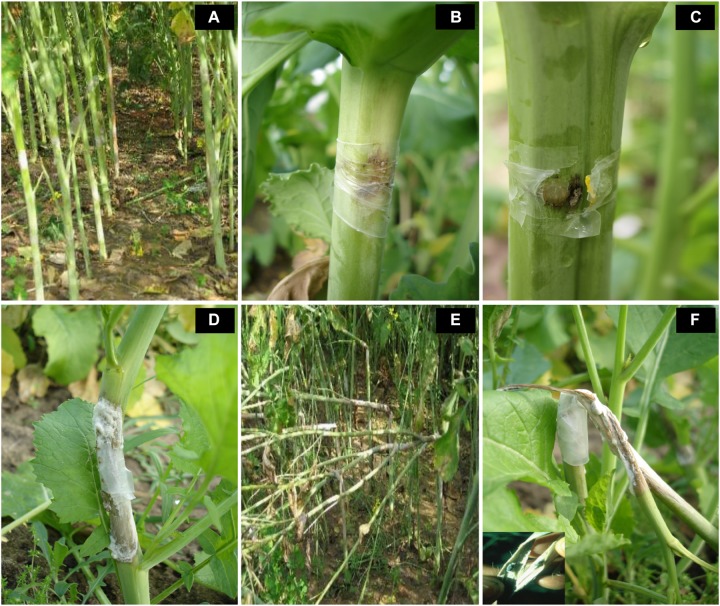
Variation in resistance responses of *B. juncea–E. cardaminoides* introgression lines, three weeks after stem inoculation with *Sclerotinia sclerotiorum*. **(A–C)** reflect highly resistant reaction. Susceptibility of recipient parent and some introgression lines was indicated by a soft watery lesion **(D)** or stem breakages due to very long lesions that girdled the stem **(E,F)**.

**FIGURE 4:**
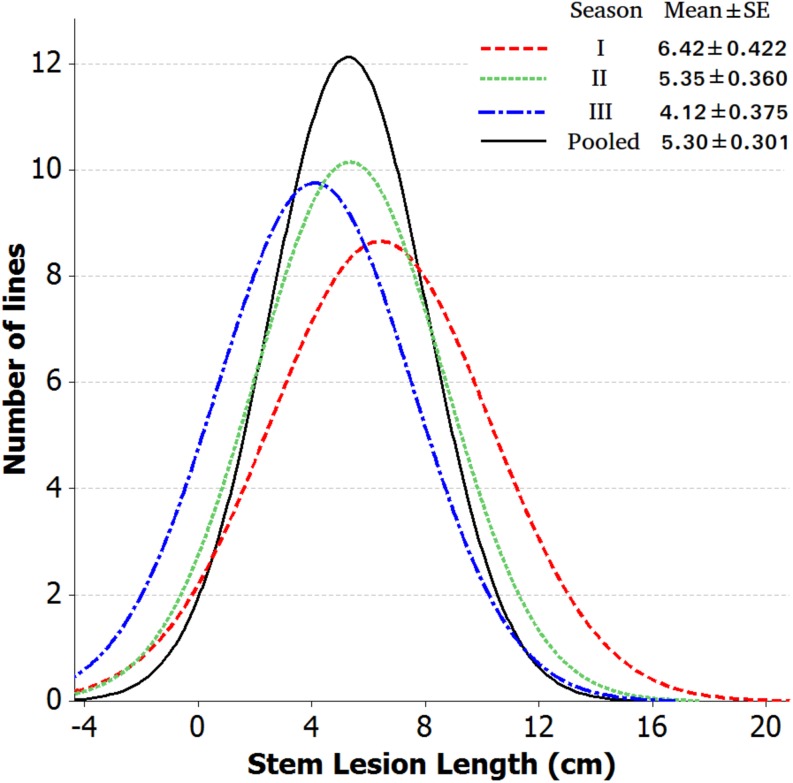
Frequency distribution of stem lesion length data of 84 *B*. *juncea–E. cardaminoides* introgression lines across each year and pooled.

Introgression lines were morphologically similar to the natural *B. juncea* cv. RLC1, except for a delayed flowering (7–10 days) and thicker main stem (Figure 1). Pollen grain stainability was used as an index of pollen grain fertility. It ranged from 75 to 95% (Figure 1), with majority of ILs clustering around 85%. IL-43, IL-45, IL-55, IL-56, IL-63, IL-92, IL-98, IL-110, IL-111, IL-124, IL-130, IL-202, etc. had very high pollen grain fertility. Normal seed set was obtained following bag selfing. All the ILs used for the present studies were first confirmed for chromosome number expected for *B. juncea* (2*n* = 36), with 18II during meiotic metaphase. GISH studies with mitotic spreads confirmed the presence of all chromosomes of both the genomes (20A + 16B). As an example, we demonstrated large introgressions for two A genome chromosomes and one B genome chromosome in two ILs using GISH (Figure 2). The ILs and their parent genotypes were assayed for their resistance responses following stem inoculation with *S. sclerotiorum*. These varied across genotypes and crop seasons. Resistant ILs showed hypersensitive response or formed a very small sized lesion (Figures 3A–C). Mean lesion length in susceptible genotype, RLC1 ranged from 7.5 to 25 cm with complete stem breakage (Figures 3D–F). *E. cardaminoides* appeared completely resistant. Analysis of variation (Table 1) showed highly significant differences among ILs for their resistance responses. Variation across years and year × genotype interactions were also significant. Replication effects were non-significant but replication × genotype effects were significant (*P* < 0.05). Mean stem lesion lengths (cm) for seasons I, II and III were: 6.42 ± 0.422, 5.35 ± 0.360, and 4.12 ± 0.375, respectively. The frequency distributions were mostly normal (Figure 4), with marginal bias toward resistant class during season III. Sixty four out of 96 ILs expressed significant resistance responses in season I, 71 in season II and 39 in season III. Of 64 resistant ILs identified during season I, 8 (IL-65, IL-83, IL-155, IL-204, IL-210, IL-215, IL-413, and IL-447) showed HR with stem lesion length <2.5 cm. Twenty three ILs showed resistance with stem lesion lengths varying between 2.5 and 5.0 cm.15 ILs expressed hypersensitive response during season II. Notable among these were (IL-17, IL-27, IL-43, IL-55, IL-83, IL-92, IL-95, IL-204, IL-210, IL-229, IL-310, IL-315, IL-413, IL-421, and IL-447). During season III, IL-65, IL-83, IL-92, IL-204, IL-215, IL-900, and IL-913 showed the best responses. Overall, IL-65, IL-83, IL-IL-204, IL-215, IL447, IL-899, IL899, IL900, IL-901, IL-902, and IL-903 were consistent in their resistant reactions over three crop seasons.

**TABLE 1:** Analysis of variance for trait stems lesion length (cm) in *Brassica juncea–Erucastrum cardaminoides* introgression lines.

**Source**	**Sum of squares**	**d.f.**	**Mean square**
Year	441.92	2	220.959^∗∗∗^
Replication	2.06	1	2.058
Block	23.92	6	3.987
Genotype	3737.89	83	45.035^∗∗∗^
Year × replication	27.21	2	13.605
Year × genotype	2442.13	166	14.712^∗∗∗^
Replication × genotype	737.80	83	8.889^∗∗^
Error	798.87	160	4.993
Total	22415.82	504	

*^∗∗^*p* < 0.01, ^∗∗∗^*p* < 0.001.*

### Association Mapping Based on SSR Genotyping

This analysis was carried on the basis of resistance responses for first 2 years. Population structure was established on the basis of SSR markers. Δ*k* was 3, suggesting the division of ILs into three groups (Supplementary Figure S1). Group I comprised of 20 ILs, whereas groups II and III included 41 and 33 ILs each, respectively. Group I mostly comprised HR or resistant (R) ILs. In contrast, groups II and III carried a mixture of MR, S, and HS ILs.

Associations between 342 marker loci and resistance against *S. sclerotiorum* were determined by MLM method as it was the best fitted model for the study. The markers with threshold −log10 (*P*) value > 2.5 were considered to be significantly associated with resistance to *S. sclerotiorum*. Primer sequences for significant SSRs are included as Supplementary Table S1. The Manhattan plot was constructed (Supplementary Figure S2). Significant markers associated with disease resistance were identified in the two environments (seasons I and II). A total of 14 marker loci appeared to be significantly associated (*P* < 0.05) with resistance to *S. sclerotiorum* in at least one season. These included 6 and 8 markers involving A and B genomes (Table 2). For A genome, six marker loci were detected in season I and five in season II. Four associations were detected when data were pooled over both seasons. Of these, three significant associations were common across both seasons as well as when data were pooled. In the case of the B genome, six associations were observed during season I, five in season II and 5 when data were pooled over both seasons. The range of phenotypic variation explained by marker loci in the B genome varied from 12.64 to 48.64% in season I and from 1.36 to 31.26% during season II. Further, for the gene/genome annotations study, the BAC sequences carrying significant marker loci were fed as queries into software MEGANTE^7^. Of the associations, involving the A genome markers, cnu_m292, cnu_m276, cnu_m418 and nia_m050 indicated association with known resistance genes. Of the associations, involving the B genome markers, Ni3H07, SB2131A, SB3751, and SJ4933 showed biological relevance.

**TABLE 2:** Significant MTAs of SSR molecular markers identified against *Sclerotinia* stem rot.

**Genome**	**Marker**	**Chr.**	**Chr. position (cM)**	**Season I**		**Season II**		**Pooled**	
				
				**−Log10^a^**	**RsqMarker^b^**	**−Log10^a^**	**RsqMarker^b^**	**−Log10^a^**	**RsqMarker^b^**
**A**	**nia_m050**	A08	69.0	3.8279	0.1676	3.8037	0.1515	4.6	0.2098
	**cnu_m157**	A09	31.2	2.6575	0.1048	–	–	4.0234	0.1683
	**cnu_m292**	*UnMp*	–	2.5528	0.0987	2.7212	0.1082	8.3768	0.561
	**cnu_m276**		–	2.8239	0.1173	2.7212	0.1396	7.8841	0.0546
	**cnu_m418**		–	2.6575	0.1013	4.1724	0.1881	–	–
	**cnu_m468**		–	4.6732	0.2063	3.8833	0.1665	–	–
**B**	**SJ4933**	B01	27.8	3.1654	0.1264	–	–	3.3553	0.1174
	**Ni3H07**	B03	164.4	6.8319	0.3339	3.266	0.1372	6.87	0.3172
	**SB2131A**	B04	0.00	8.5622	0.4864	6.259	0.3126	–	–
	**SJ1505**	B06	59.2	3.7539	0.1767	–	–	7.366	0.0294
	**SB1728**	B08	24.8	3.178	0.1328	2.9208	0.0136	–	–
	**SB3751**		52.0	6.676	0.3285	4.6879	0.2123	3.4368	0.6618
	**Ni2A09**	*UnMp*	–	–	–	5.1811	0.076	–	–
	**Ni4C09**		–	–	–	–	–	5.1156	0.2351

*A total of 14 marker loci were identified to be significantly associated with *Sclerotinia* resistance in at least one environment. These included 6 for A-genome and 8 for B-genome. ^*a*^Negative logarithm value of *P*-value of each significant marker; ^*b*^Phenotypic variation (%); *UnMp* – un-mapped marker.*

### GWAS Based on SNP Genotyping

**FIGURE 5:**
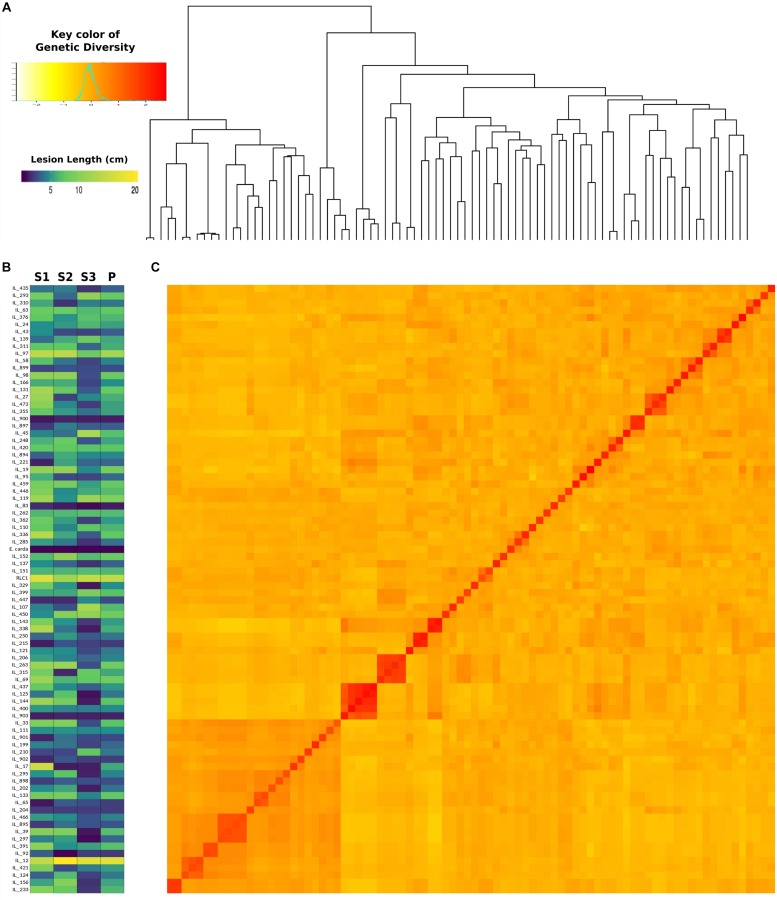
Heatmap showing resistance responses of 84 *B*. *juncea–E. cardaminoides* ILs, susceptible parent (RLC1) and resistance donor species *E. cardaminoides* over three crop seasons **(A),** Hierarchical clustering of ILs based on their SNP genotypes **(B)**, kinship heatmap based on genetic distance between ILs **(C)**.

**FIGURE 6:**
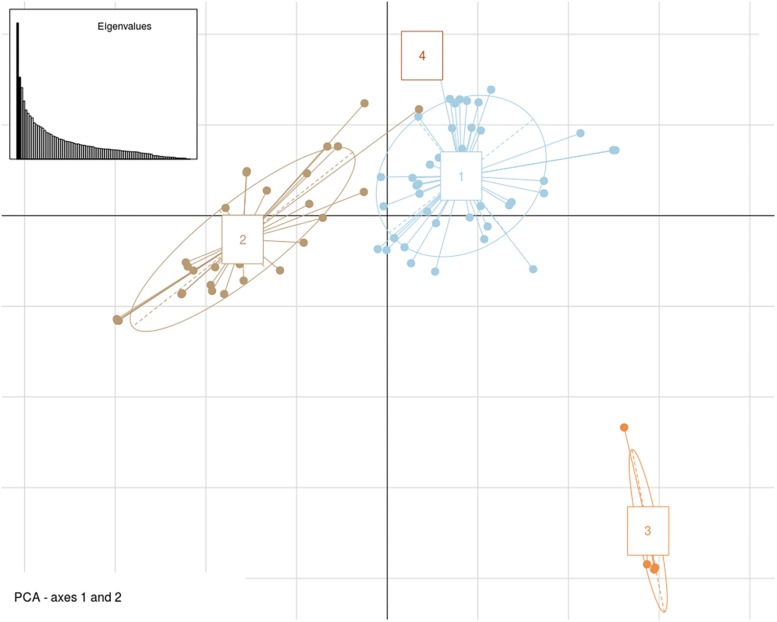
Genetic structure patterns of 84 *B*. *juncea–E. cardaminoides* introgression lines distributed into 4 groups after correction for discriminant analysis of principal components (DAPC).

Genome-wide association analysis was carried out by using transformed resistance rank values of 84 genotypes and 78,578 SNPs (MAF > 0.10). These markers were spread across all 18 chromosomes of *B. juncea* (Supplementary Figure S3). Kinship matrix and covariates (PCA) data was generated through “MVP.Data” function of the software MVP.r^8^. This was used to adjust for the confounding effects of population structure and kinship. Horizontal lines on top of heat map show hierarchical clustering of ILs (Figure 5A). There were three broad groups, of which first group seemed most diverse and ILs included in this group showed consistently superior resistance responses. This is shown in the heat map of resistance responses of ILs in terms of lesion length (cm) following stem inoculation with *S. sclerotioum* over three seasons (Figure 5B). Heat map of kinship matrix showing genetic relatedness among 84 ILs is available in Figure 5C. DAPC was implemented in R software package “Adegenet.” It also showed three clear groups (Figure 6). These broadly confirmed inferences drawn from SSR data. PC and DAPC were used as covariates in different GWAS analysis algorithms to reduce false positives by minimizing the effects of population stratification.

**FIGURE 7:**
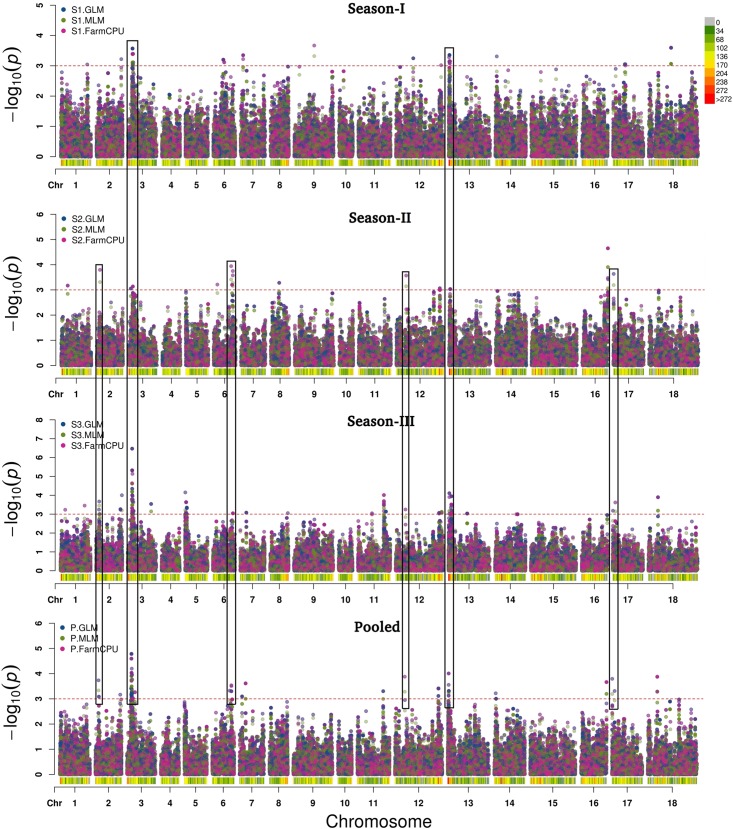
Manhattan plots after GWAS analysis based on 78,578 SNPs for trait stem lesion length at threshold level [–log10(*P*) > 3.0]. SNPs in black rectangles depicted strong MTAs over the seasons.

GLM, MLM, and FarmCPU methods were implemented in the software MVP.r. Software default settings were primarily used to identify MTAs. Analysis yielded a large number of significant findings. These were subsequently confirmed using alternate algorithms as implemented in tassel and adegenet. We considered only those MTAs-that were consistent across at least two algorithms. Marker positions were same in primary GWAS software, the validating algorithm and the season. Also included were markers consistently detected over at least two seasons (Table 3 and Figure 7). Blast2GO Pro was used for annotating 50 kb regions (25 kb each on both side of identified SNP). In total, 55 SNPs were significantly (*P* < 0.001) associated with resistance to *Sclerotinia* stem rot. MTAs were mainly located on chromosomes A03, A06, B03 and B04.

**TABLE 3:** The list of significant SNPs identified in consensus over seasons and different algorithms along with SNPs rich annotation information.

**Chr**	**SNPs**	**SNP ID**	**Marker Interval**	**PVE (%)**	**−log10(*p*)**	**SNPs consensus over**		**Annotation**		**Description**
								
						**Seasons**	**Algorithms**	**Gene Name (distance from SNP in kb)**	**Gene Bank Identifier (NCBI)**	
**A03**	2	A03_6235895, A03_6236020	6235895-6020	17.01	4.34	S2 + S3 + P	FarmCPU, MLM, GLM(T), MLM(T)	SBT4.4 (0.01); SBT4.9 (0.04); SBT4.12 (0.12)	75170491; 30793835; 332009758	Subtilase family protein
	11	A03_6274763, A03_6274795, A03_6274819, A03_6274834, A03_6274836, A03_6274844, A03_6274857, A03_6274863, A03_6274875, A03_6274939, A03_6274994	6274763-994	18.33	4.22	S1 + S3 + P	FarmCPU, MLM, GLM(T), MLM(T)	At1g65850 (23.43); At3g04220 (23.29); At5g11250 (24.24)	334183667; 1039014440; 1039021411	Disease resistance protein (TIR-NBS-LRR class) family
	3	A03_6337002, A03_6337034, A03_6337047	6337002-047	12.58	3.38	S1 + S3 + P	FarmCPU, GLM(T)	GSTT2 (3.39); GSTT3 (3.39)	332007273; 62321525	Glutathione S-transferase THETA 3
	5	A03_6390210, A03_6390240, A03_6390303, A03_6390342, A03_6390381	6390210-381	13.36	3.46	S1 + S3 + P	FarmCPU, MLM, GLM(T), MLM(T)	At5g16590 (18.08)	75171650	Leucine-rich repeat protein kinase family protein
**A06**	10	A06_14196946, A06_14196963, A06_14196967, A06_14196987, A06_14197016, A06_14197024, A06_14197037, A06_14197041, A06_14197047, A06_14197052	14196946-7052	13.66	3.24	S1 + S3	FarmCPU, GLM(T)	BSK4 (7.79); BSK7 (7.79); BSK8 (7.79)	1352911964; 1352911965; 75333858	Kinase with tetratricopeptide repeat domain-containing protein
	5	A06_26227171, A06_26227260, A06_26227356, A06_26227690, A06_26227735	26227171-7735	13.98	3.25	S2 + S3 + P	FarmCPU, GLM(T), MLM(T)	AOC3 (0.21); AOC2 (0.11); AOC1 (0.25); AOC4 (0.21)	73921673; 7939564; 73921671; 34391988	Allene oxide cyclase
	5	A06_27175971, A06_27176024, A06_27176029, A06_27176035, A06_27176193	27175971-6193	15.59	3.48	S2 + S3 + P	FarmCPU, MLM, GLM(T), MLM(T)	LCR73 (3.64); PDF2.1 (3.62); PDF2.3 (3.61); PDF2.5 (3.64); LCR71 (3.61); LCR76 (3.64)	46396253; 15226876; 15226878; 15242860; 254763270; 79323842	Protease inhibitor II
**B03**	4	B03_924478, B03_924540, B03_924955, B03_925003	924478-5003	14.39	3.39	S1 + P	GLM(T)	SBT3.3 (21.80)	34098815	Subtilase family protein
	9	B03_998752, B03_998785, B03_998792, B03_998793, B03_998812, B03_998815, B03_998818, B03_998821, B03_998898	998752-898	15.16	3.45	S1 + P	FarmCPU, GLM(T)	PBL7 (22.07)	122230074	Protein kinase superfamily protein
**B04**	1	B04_1922364	1922364	15.73	3.41	S1 + S3 + P	FarmCPU, GLM(T), MLM(T)	At1g65850 (4.29); At3g04220 (4.29); At5g11250 (7.73)	334183667; 1039014440; 1039021411	Disease resistance protein (TIR-NBS-LRR class) family

Two SNPs (A03_6235895 and A03_6236020), present on chromosome A03 explained 17% of trait variation. The closest genes (*SBT4.4; SBT4.12*, and *SBT4.9*) encode proteins belonging to the subtilase family. Another group of eleven SNPs were identified in a small genomic region on the same chromosome (6,274,763-994 comprising 231 bp). Genes closest to this genomic region were *At1g65850*, *At3g04220*, and *At5g11250*. These encode disease resistance proteins of class TIR-NBS-LRR. Also located closely on the same chromosome were *GSTT2* and *GSTT3*. These encode glutathione S-transferase *THETA 3*. Chromosome A03 also harbored a group of five SNPs (A03_6390210, A03_6390240, A03_6390303, A03_6390342, and A03_6390381). Annotation in the region was suggestive of the leucine rich repeat protein kinase family protein. *At5g16590* was indicated in close genomic proximity. Twenty SNPs were identified on the chromosome A06. Of these 10 were within a genomic interval of 102 bp (14,196,946-7,052). Annotation indicated involvement of *BSK4*, *6*, *7* and *8* and kinase with tetratricopeptide repeats domain protein. Five SNPs were identified between intervals of 26,227,171-7,735, close to allene oxide cyclase (*AOC1-4*). Another group of five SNPs (A06_27175971, A06_27176024, A06_27176029, A06_27176035, and A06_27176193), were present near protease inhibitor 2 (*LCR71*, 73, 76; PDF 2.1, 2.3, 2.5). Thirteen SNPs were localized on two genomic regions on chromosome B03. Of these, four SNPs were present in the genomic interval between 924,478-5,003. Annotation indicated the presence of gene *SBT3, 3*, encoding subtilase family protein. Nine SNPs (998,752-898) were positioned close to *PBL7*, encoding protein kinase superfamily protein. One SNP (1,922,364) situated on chromosome B04, appeared to be present close to *ARM* repeat superfamily protein (*PUB4, 10, 11*). The identified SNPs explained 12–16% of the phenotypic variation. Flanking sequences of candidate SNPs extracted from mock-up pseudomolecules reference are included in Supplementary Table S2.

### SNP Validation

Six peak SNPs, significantly associated with resistance responses were chosen for validation using sanger sequencing. PCR products were amplified from eight ILs, varying for their resistance responses. Sanger sequencing and sequence alignment validated five SNPs out of the six sampled (Table 4).

**TABLE 4:** Validation of trait associated peak SNPs.

**Significant SNP**	**SNP**	**Forward Primer**	**Reverse Primer**	**Validated**
A03_6235895	G/A	5′CACACCTCTCTCCCACGATCTC3	5′GCAATCACACACGATGGTCA3′	Validated
A03_6390210	C/A	5′AGTTCATTGCCGTTGTTGCT3′	5′AAGCTGATAAGAGGCGTCGA3′	Validated
A06 14197052	A/G	5′TGAGCTTTTCCTTCCCTGCT3′	5′GGCAGTGTTTGGGAATGAGA3′	Failed
B03_998898	G/T	5′GAGGAAGAGCAGTAAAAGCATC3′	5′AGACGCGTACAAGAGTTCCT3′	Validated
B03_924478	C/T	5′CGTTGCTCCGATCAGGTCAG3^″^	5′ATCCCACTCACATCTCCACC3′	Validated
	T/C	5′CGTTGCTCCGATCAGGTCAG3′	5′ATCCCACTCACATCTCCACC3′	Validated

### Intersection of MTAs Identified Using SSR and SNP Genotyping

**FIGURE 8:**
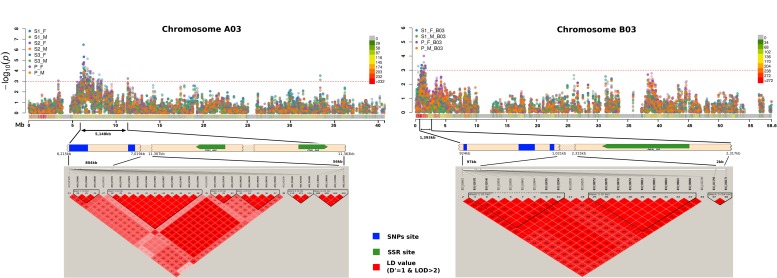
Chromosome wise Manhattan plots (top) for stem lesion length trait. Vertical bar below Manhattan plot areas shows SNPs hotspot for trait identified by association mapping. Linkage disequilibrium (D′ value) matrices (bottom) are plotted for regions denoted by anchoring lines. Regions of strong LD of SNPs site (blue) and SSR maker site (green) are shown in red. Significant association markers are denoted plotted above the threshold (doted redlines). ∼0.8 Mb **(A)** and ∼97 kb **(B)** regions show strong MTAs on chromosome A03 and B03, respectively.

Linkage disequilibrium (D′ value) matrices were plotted for chromosome regions (A03, B03) showing multiple SNPs (Figure 8). Strong LD blocks were indicated by the occurrence of adjacent loci in LD. Regions of strong LD of SNPs and SSR maker sites were recorded. Major LD blocks co-localized with the regions showing strong MTAs in chromosome A03 and B03, respectively. SSR markers, namely cnu-468 and nia-50, showing association with resistance responses (Table 2), were in strong LD with SNPs showing MTAs in chromosomes A03 and B03.

## Discussion

Introgression of alien genomic fragments bearing the genes of interest from wild *Brassicaceae* species is a major tool to broaden the genetic base in crop Brassicas. In spite of its potential applications, this method of germplasm enhancement is rarely used, possibly due to the difficulties involved in initial steps of inter-specific or generic hybridizations and time required to stabilize introgressions in the host genome(s). Linkage drag associated with the introgressed variation is another limitation as the size of random introgressions can be highly variable with uneven distribution (Curtis and Lukaszewski, 1993). The drag can be reduced by inducing recombinations between alien and crop genomes. Physical disruption of introgressed alien chromosome fragments through heavy irradiation and then identifying plants with reduced size of introgressed genomic fragment through low pass sequencing is now a feasible option. A major prerequisite to such an approach is the information regarding the chromosome fragments responsible for the trait variation and their location on the chromosome(s). Toward that end, we report our success in mapping genomic regions responsible for the stem resistance to *S. sclerotiorum* in *B. juncea*–*E. cardaminoides* ILs.

Introgression lines showed varied resistance responses with a near normal distribution, implying a quantitative inheritance. Kinship and population structure analysis of ILs identified three distinct clusters. HR genotypes grouped with the resistance donor species *E. cardaminoides*. Association mapping using SSR genotypes allowed identification of six marker loci on A and B genomes. This information was reinforced though GWAS based on genotyping by sequencing. Multiple MTAs, occurring in a very close proximity, and/or adjacent regions, were repeatedly identified on chromosomes A03, A06, B03 and B04. Chromosomes A03 and A06 harbored the maximum number of resistances associated SNPs in present investigations. It is likely that a greater homology between A and E^C^ genomes (Chandra et al., 2004) allowed preferential introgression of resistance bearing chromosome fragments from *E. cardaminoides*, on the A genome of *B. juncea*. As explained earlier, we confirmed three large *E. cardaminoides* segment substitutions in two ILs through GISH. Two introgressions were located on A genome chromosomes and one on B genome chromosome. GISH can only detect large translocation/alien chromosome segment substitutions. However, many smaller genomic exchanges must have occurred between A/B and E genomes due to homoeologies that exist between three genomes (Chandra et al., 2004). ILs are also likely to vary for number and type of alien introgressions. The introgressions are generally dependent on homologous regions for recombination or random translocations. Annotation of associated genomic regions in our studies highlighted an array of resistance mechanisms in terms of signal transduction pathways, hypersensitive responses, oxidative burst and production of anti-fungal proteins and metabolites. Up regulation of many of these genes have been reported following *Sclerotinia* infestation in *B. napus* (Rimmer et al., 2007). There is no previous report for QTL mapping for *Sclerotinia* stem rot resistance in Brassica crops. However, mapping in euploid *B. napus* has shown resistance associated QTLs on chromosomes A06 and A08 (Zhao et al., 2006; Li et al., 2015; Wei et al., 2016). Wei et al. (2016) elucidated resistance genes and pathogenesis related genes through GWAS of 347 accessions of *B. napus*. They could identify 17 significant associations for stem resistance on chromosomes A8 and C6. In line with the previous reports, our studies also highlighted the genetic complexity of resistance responses to *S. sclerotiorum*.

Eleven SNPs (A03_6274763-994) were identified close to three R-genes, encoding *TIR-NBS-LRR* proteins, at a distance of 23.29–24.24 kb. This protein family constitutes a second line of defense and is involved in the detection of specific pathogen signals such as avirulence (*Avr*) factors; mediating physical association between resistance proteins and pathogen effector molecules; activation of signal transduction pathways and as a consequence up regulation of many defensive proteins and compounds. SSR marker Cnu_m276 was also associated with leucine-rich repeats (*LRR*) disease resistance proteins (Chen et al., 1998; Pilet, 1999). *LRR* proteins include tyrosine kinase receptors, cell-adhesion molecules, virulence factors and extracellular matrix-binding glycol-proteins. The most studied plant signaling *RLK* is *LRR-RLK* Brassinosteroid. Insensitive 1(*BRI1*) mediates signaling which includes phosphorylation of various members of brassinosteroid signaling kinases (*BSKs*) with tetracopeptide repeat domain by *BRI1* (Gruszka, 2013). In the present study, 10 SNPs (14,196,946-7,052, explaining 13.66% variation) may be associated with three *BSKs* genes on chromosome A06. Brassinosteroid signaling is involved in many cellular processes; like increased accumulation of reactive oxygen species (ROS) known as oxidative burst (Kim et al., 2012), triggering enhanced production of defensive proteins and metabolites, including peroxidases, protease inhibitors, and *AOC*. MTAs (9SNPs) involving protein kinase superfamily protein were identified on chromosome B03. Members of this family are involved in defensive responses to abiotic stress and pathogen invasion (Cheng et al., 2002). Another defensive protein was recognized through five SNPs, present very closely (at a distance of 110–250 bp) to four candidates encoding functional *AOC* polypeptides (*AOC*1-4) on chromosome A06. *AOC* catalyzes the essential steps in biosynthesis of jasmonic acid (Stenzel et al., 2012), a mainstay of signaling pathways during plant stress responses. Mutants defective in these genes were vulnerable to pathogen invasion (Park et al., 2002; Von Malek et al., 2002; Browse, 2009). Chromosome A06 also harbored five SNPs within a small genomic interval (27,175,971-6,193). Candidate genes closest (3.6 kb) to SNPs encode protease inhibitor II, an anti pest metabolite (Juge and BirteSvensson, 2006) which inhibits pathogen proteases and deters their invasion. Glutathione S-transferase *THETA3* (GSTT3) (A03) – predominantly catalyzes reduction of organic hydroperoxides formed during oxidative (Dixon et al., 2002). Multiple SNPs located in a small genomic region(s) on a given chromosome can be construed as constituents of major QTLs.

Marker trait associations involving two SNPs (A03_6235895 and A03_6236020) on A03 and four SNPs (B03_924478-5003) seemed important to explain *E*. *cardaminoides* resistance. The closest candidates (*SBT 3.3*, *SBT4.4*, *SBT4.12*, and *SBT4.9*) encode subtilase family proteins, which are critical for signaling cascades during pathogen recognition, immune priming and petal and stamen development (Figueiredo et al., 2014). *SBT 3.3* is a regulator of primed immunity (Ramírez et al., 2013). It is possibly a plasma membrane receptor, activating downstream immune signaling processes. Some subgroups of plant subtilisin-like proteases, may play a role, similar to caspases in animal programmed cell death (PCD) (Chichkova et al., 2010; Vartapetian et al., 2011). Pathogen recognition results in growth inhibition, which may trigger a hypersensitive reaction, a form of localized PCD. This is central to innate immune responses (Vartapetian et al., 2011). A positive feedback loop circuit likely maintains the *SBT3.3* expression, after initiation of signaling process and maintenance of expression threshold levels may be important to keep cells in a sensitized mode (Ramírez et al., 2013). Three SNPs (A03_6337002-047) appeared linked with GSTT3 belonging to RING/U-box super family protein is in a close genomic proximity. SSR marker locus cnu_m418 was also associated with F-box related proteins (Kipreos and Pagano, 2000; Jain et al., 2006). They serve as positive regulators of ETI responses, which are required for full plant resistance to avirulent pathogens (Marino et al., 2012). Occurrence of SNPs or group of SNPs encoding multiple resistance genes in a region spanning approximately 885 kb are on chromosome A03 and 74 kb on B03 was particularly significant as these confirmed the past arguments suggesting clustering of resistance genes in the genome (Graham et al., 2002).

Summarizing, we demonstrate the successful introgression of chromosome fragments from *E. cardaminoides*. Given the quantitative nature of *cardaminoides* resistance, it is likely that some of the identified MTAs may involve small effect resistance genes already present in *B. juncea*. However, significantly higher level of resistance in ILs suggests that bulk of these must come from wild donor species. Linking of identified MTAs unequivocally to *E. cardaminoides* should await comparison of its genome sequence with those of ILs. We are also developing *E. cardaminoides* specific oligo probes to cytogenetically map all ILs for introgression sites. For future research, we are planning to undertake RNA-Seq data analysis to study transcriptomic responses to pathogen infestation in *B. juncea, E. cardaminoides* and selected *B. juncea*–*E. cardaminoides* ILs. These are expected to help in identifying key genes that define *cardaminoides* resistance to *Sclerotinia* stem rot. Host resistance is the only avenue for long-term, cost-effective management of this devastating, worldwide pathogen of *Brassicas*. Our research has clearly opened the way for deployment of the introgressed gene(s) into a wide range of high-yielding cultivars, of *B. juncea* initially and, subsequently, into other crop and horticultural *Brassica* species.

## Data Availability

All critical data, like flanking sequences of peak SNPs, is included in the manuscript or the Supplementary Files. Genotyping and phenotyping data supporting the conclusions of this manuscript will be made available by the authors, without undue reservation, to any qualified researcher.

## Author Contributions

SB and MB designed the research. SB and CA constructed the introgression lines. KR, AG, and PS performed the stem inoculations and recorded the disease score. CA, RK, AS, and MS maintained the germplasm and isolated the DNA. JA and NK conducted the bioinformatic analysis. KR and SB interpreted the data and wrote the manuscript. GK validated the SNPs. MB edited the manuscript. SB supervised the whole study. All authors read and approved final version of the manuscript.

## Conflict of Interest Statement

The authors declare that the research was conducted in the absence of any commercial or financial relationships that could be construed as a potential conflict of interest.

## Footnotes

1 http://brassicadb.org/brad/datasets/pub/Genomes/Brassica_juncea/V1.5/

2 http://bbi.irri.org/products

3 http://www.maizegenetics.net

4 https://megante.dna.affrc.go.jp/

5 https://rdrr.io/github/XiaoleiLiuBio/MVP/

6 http://adegenet.r-forge.r-project.org/

7 https://megante.dna.affrc.go.jp/

8 https://github.com/XiaoleiLiuBio/MVP

## Supplementary Material

FIGURE S1 Structure Analysis of introgression lines. Δ*k* = 3 thus the division of introgression lines in 3 groups.

FIGURE S2 Manhattan plots in Season I, II and Pooled over seasons.

FIGURE S3 Genome-wide association reveals markers distribution in all 18 chromosomes of *B. juncea.*

TABLE S1 Primer sequences of significant SSR markers used in the studies.

TABLE S2 Flanking sequences of candidate SNPs extracted from mock-up pseudomolecules reference (SNP base denoted by bold and underline character).
