# Supplementary figures and images for: Detection of First Marker Trait Associations for Resistance Against Sclerotinia sclerotiorum in Brassica juncea–Erucastrum cardaminoides Introgression Lines
Source: Front Plant Sci. 2019 Aug 6;10:1015. doi: 10.3389/fpls.2019.01015 (PMC6691357; doi:10.3389/fpls.2019.01015)

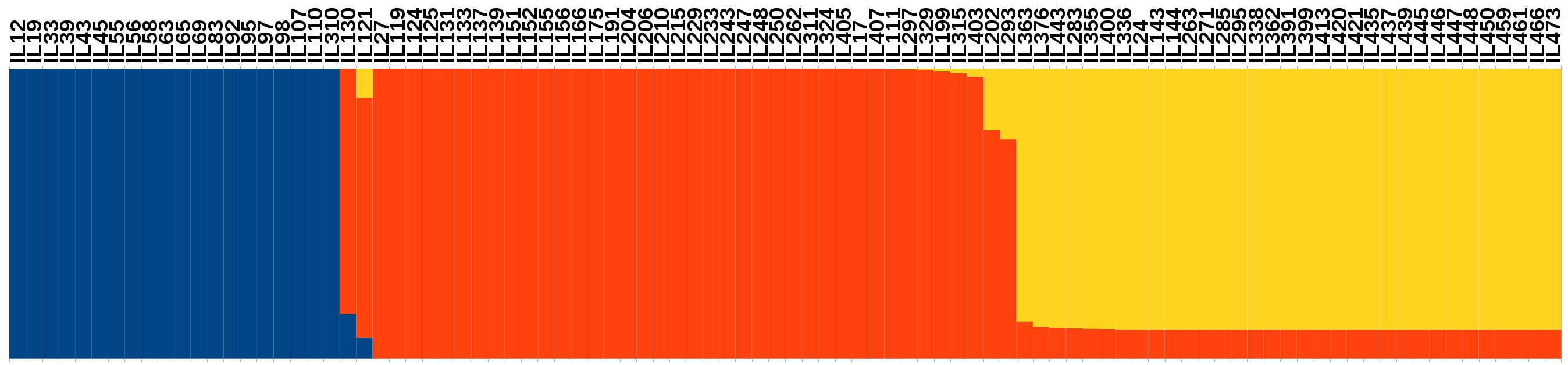

Supplement: FIGURE S1 — Structure Analysis of introgression lines. Δk = 3 thus the division of introgression lines in 3 groups. [file Image_1.TIF]

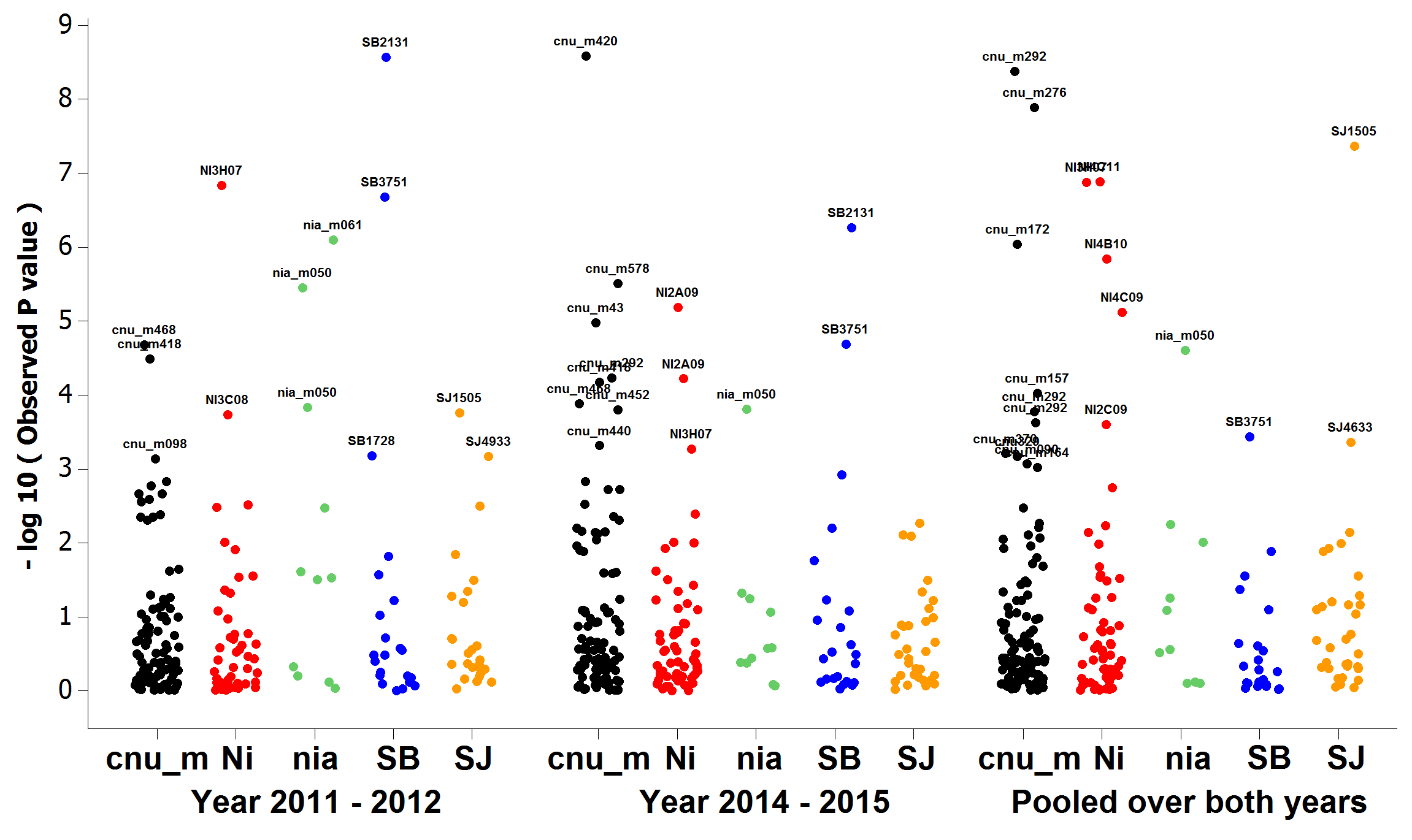

Supplement: FIGURE S2 — Manhattan plots in Season I, II and Pooled over seasons. [file Image_2.TIF]

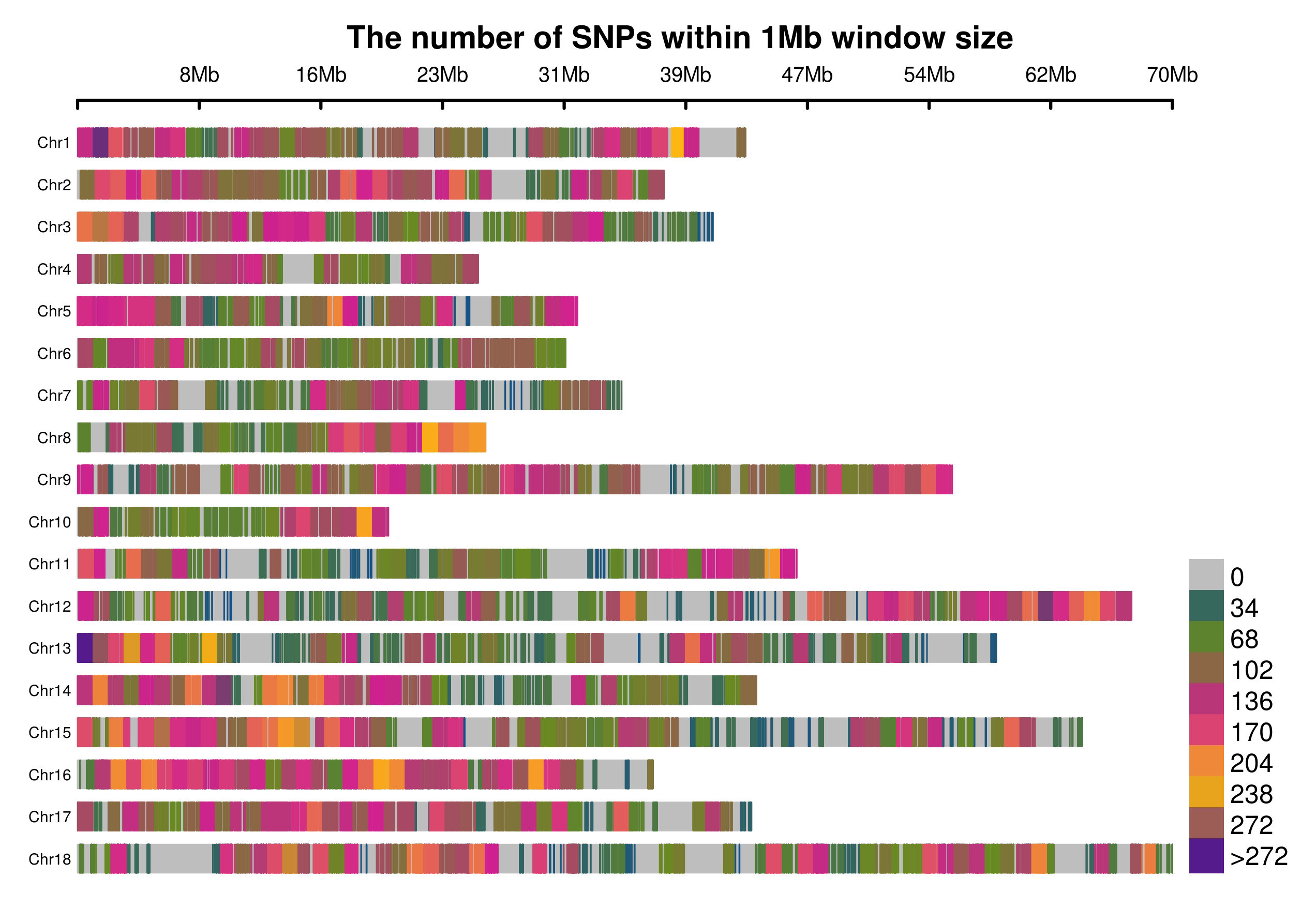

Supplement: FIGURE S3 — Genome-wide association reveals markers distribution in all 18 chromosomes of B. juncea. [file Image_3.TIFF]
