# Supplementary material for: Detection of First Marker Trait Associations for Resistance Against Sclerotinia sclerotiorum in Brassica juncea–Erucastrum cardaminoides Introgression Lines
Source: Front Plant Sci. 2019 Aug 6;10:1015. doi: 10.3389/fpls.2019.01015 (PMC6691357; doi:10.3389/fpls.2019.01015)
Supplement: TABLE S1 — Primer sequences of significant SSR markers used in the studies. [file Table_1.docx]

Supplementary Material

**Detection of first marker trait associations for resistance against *Sclerotinia sclerotiorum* in *Brassica juncea−Erucastrum cardaminoides* Introgression Lines**

**Rana, K., Atri, C., Akhatar, J., Kaur, R., Goyal, A., Singh, M.P., Kumar, N., Sharma, A., Sandhu, P.S., Kaur, G., Barbetti, M.J. Banga, S.S.***

*** Correspondence:** Corresponding Author: Surinder S. Banga [nppbg@pau.edu](mailto:nppbg@pau.edu)

**Supplementary Table 1**. Primer sequences of significant SSR markers used in the studies.

| **S.N.** | **Genome** | **SSR Primer** | **Forward primer sequence** | **Reverse primer sequence** |
| --- | --- | --- | --- | --- |
| 1 | A | nia_m050a | GCCATGTGGCCTTCTAAAAA | CGATTCCTGGTCATTTGGAT |
| 2 |  | cnu_m157a | CCGCAGTTGATCCATTAGCC | ACGCTGCATCCACATGAAAC |
| 3 |  | cnu_m292a | TGGATGATAAGGTCAACCACA | ATCGAACCTGGGTCTAAGCA |
| 4 |  | cnu_m276a | CAGCTGCAGGCTTAACAGAGC | TGTGGTGCCATGACCAGTCT |
| 5 |  | cnu_m418a | AAGTGGGCTTTGTTGTGGTT | CGACGGATTAAGGCTTTTGA |
| 6 |  | cnu_m468a | CTGAAGCTTCCTCCGACAAC | TAACGAGATCGGCGAAGAAT |
| 7 | B | SJ4933 | Sequence obtained under MTA from Isobel Parkin, Agri-Canada | |
| 8 |  | SB2131A |  |  |
| 9 |  | SJ1505 |  |  |
| 10 |  | SB1728 |  |  |
| 11 |  | SB3751 |  |  |
| 12 |  | Ni3H07 | GCTGTGATTTTAGTGCACCG | AGCCGTTGATGGAATTTTTG |
| 13 |  | Ni2A09 | CGCGAGTAAATCAATGTGAATC | CGACCCACCAACTCACTAAC |
| 14 |  | Ni4C09 | AGCATCAATCTTTTGCTCTGC | TGCACACAAACTCCTTCTCC |
