# Supplementary material for: Detection of First Marker Trait Associations for Resistance Against Sclerotinia sclerotiorum in Brassica juncea–Erucastrum cardaminoides Introgression Lines
Source: Front Plant Sci. 2019 Aug 6;10:1015. doi: 10.3389/fpls.2019.01015 (PMC6691357; doi:10.3389/fpls.2019.01015)
Supplement: TABLE S2 — Flanking sequences of candidate SNPs extracted from mock-up pseudomolecules reference (SNP base denoted by bold and underline character). [file Table_2.docx]

Supplementary Material

**Detection of first marker trait associations for resistance against *Sclerotinia sclerotiorum* in *Brassica juncea−Erucastrum cardaminoides* Introgression Lines**

**Rana, K., Atri, C., Akhatar, J., Kaur, R., Goyal, A., Singh, M.P., Kumar, N., Sharma, A., Sandhu, P.S., Kaur, G., Barbetti, M.J. Banga, S.S.***

*** Correspondence:** Corresponding Author: Surinder S. Banga [nppbg@pau.edu](mailto:nppbg@pau.edu)

**Supplementary Table 2.** Flanking sequences of candidate SNPs extracted from mock-up pseudomolecules reference (SNP base denoted by **bold** and underline character).

| **S.N.** | **Fasta Sequence** |
| --- | --- |
| 1 | >A03_6235895  AATTATATATGTCATTGTTAGAATCCTCAAAATTTAAAGAGAATGATTAGAGAATGAACTAATCAAATCACACCTCTCTCCCACGATCTCTTGAAGAATACTCATGTGATGGGACATTGGTGCGTAGTCTGCTTGAGAAGGAAGTGAACCCATGTAGACGATATACTCCTTAATGCAATGAGAATCACATTTATCAATGT**G**TTCATCATTAGGAGAAGTGCACATATATTGCTAAACGCAAGAGGTTTTGATCATTTGTACCTGTTTATCTTGACCATCGTGTGTGATTGCTATGATTGAAGATGAGAACAACAAGACAAGGAGACATGAGAGGAAGCTAGGGAAAGGTCCTCGTTTCACCATCTTTAGTAATGGTAAAAGTTGGTTTATTATTTCTATGG |
| 2 | >A03_6274763  AAAGGTGTAGATTATGCATTACTTTTGTCTTGCTCAAAAAACAATGCATTACTTTTGTCAAACAGTTAGTCCTTTTCGATAATGAATTATTTATTTCGTTTTATAATCGTGTAGCGTGTTATTTATCGAAATATCCGGTCCAACTTGCTGAAGTTTTTGCAAATTGTGTTTTTGTTTGTTAAATATTATAAAGGGGTATG**G**TTCGTGCATTGTCACTTGGAAATCCATACAATTAGGGGACTAAAGATGGCATTCGCCGTCGACAGGTCACCGCCCGTGACATGTCACTGCTTCTTACACCCACCGATCTTTTCAGTGTTAGGACTTTTTTTTTGTAATTTTAGTTTATACTTAGTACCTATAAGGTGCATGGTTAGAATAATTTGCATGTAAAAAAATTT |
| 3 | >A03_6337002  AGGCAAAACCATGTATCTTAATTGTCATTTCTATGGGATCTCGTTATCACATGTAAAAAAATCTACAGCTTTGATGAGATGTGACAGAATCTCCGAGGGCTACATCACTCACTTGGCAGTTGAGGTCAAAGCATCTACTGTTTCACTCATTGTTTGCAAACTGTAAACTATGCTTTATTCTTGGCATCTTCTAGCTTTCA**T**AATCATTTCATATTCAGTAGGACACCTTTTTAGCAGTTTTATAAAAGACTAGTCGTTTTCATATAACACAGAATAAACAAAGAGATGGAGAAAACGAGTGATGAATTAAGAAAGACGTTAACTTTGTAATGACCACAACATATAAACCTATGATATTTGAGAGTTCAATCAAAGTATGAATTTTCCACATCAGATGAGCT |
| 4 | >A03_6390210  GGTTTGAACACCAATGTATACTTACTTACCAGCGATATATGGGAGCAAGAGCTATCTCCTCTAGTCTACATGGTCATCGTTGGATTCATCACTAAGTTCATTGCCGTTGTTGCTGCTGCTGTCTTCTTTAAAGTCCCTACTAGAGATAGTCTCACGTTAGGTCTC6ATGATGAATTTGAGAGGCCAGATTGATATGTTACT**C**TACTTGCATTGGATAGACAAACGTATTGTGGGTTTAGCCGGTTTTACTGTTTTGGTTTTGCAATCGCTTGTGATTACCGGTATATCGACGCCTCTTATCAGCTTCCTTTACGATCCAAACCGTCCTTACAGGATCAGCAAGCACCGCACCATCCAACACACTCCTCCTTCTACCGAGATGGGTTTGGTTCTTGCCGTGTC |
| 5 | >A06_14197052  GATCTCTCCTGACCCTGTTGTGAGTGGCAAAGCCGCAACATTTAAGATCACTGGTTCTACTGGTATGAGCTTTTCCTTCCCTGCTTCTTAAAAGCTTACAACATACATACACTCACTCTGTCAACATTTCCTTGGTGTGAATAGATGAAGACATCTCTGGAGGAGAAGTAGTCATCAGTGTTTCACTCTATGGAGTTCAT**A**TCCATACCGAAACTCATGATCTCTGCGATGAGTCGTCCTGCCCGATCGCACCTGGCACCTTTGTCCTTTCTCATTCCCAAACACTGCCTTCTATTACACCACCCGTAAGTCATCTTCCTGTTTCTCCTTGCCATCATTATCAGCTAAGGCTTCTTGACTGATCTCTCTTTGAAACAGGGTACTTATACGCTTAAGATGAC |
| 6 | >A06_26227171  AGACCGAACGTTAGGCAAGCGGCGTTTTTCAGGATGTTTGGGCTGTGTCGGTCTAGTTCGTTGAGCTCGTACACGCTCAGTTCTTGAATTCTACCTGAATTTTCAAGAACAAAGAAAATAAAAAAACGAAAACTCAATTTGTGAATTTTGAGATCATGCTTAAAAATATATGAAATGTCCTCAAAAGACAAAAAAAAAAA**A**GGATCAATGAACTTACTTGGTCTGGAGTTTTCGATATGTCCGTTCTGGGAGAGAGCTCGGGACAGAGTGAGGTTCTCGGGAGTGGAAGGTGAACGAGAGGAGAAACCCGAACCGTTGGATGAGATCCCAAGAGTCTTGAAGTGTTTAGAGAAACCGAGAAGAGAGTTACGATGAGATTGGTGGTGACAGGAGAGATTGTT |
| 7 | >A06_27176193  ATTTATATGGTTTTGGGTTTGTATGAAATTGTATTCTTATGATCAGTTATGGTTTTGGGTGTGTATGACTTGGTTTGATCTCTAATCTCACGTTTACATTTTATATGTATGTCATATTTCCTGTTTGATTAGCATTGGACCAACACAAAAACATGTCTAAAGATATTACAAAAGTACATGCCCATTCATGTATGTTTGAT**A**TTTTATATAGACGCATTCGTATAGTATGTAGATTTAGGAAGTTAATTATAAAATATTAAATTAATACATTGCTATCAGTATTTTCAAACAATTTTTTATTCTTTTTTATACTACTATCTTTATCCATGTTTCCAAACATATTTAAATGGTACTTCTACTATAATAAGATAGATAGATTTTTTAATTTGATATATATATAT |
| 8 | >B03_924478  TTCTCCAGGACGGTTTCTCGACGGCAAAACTCGGTCGTTGCTCCGATCAGGTCAGAGAAGAATGTTAAAGAAGGGACTCTAAACGACGACGTTTCGGTGGAGAGCCAGAGTCTTCAGTGGGCTCAGGGGAAAGCAGGTGAGGATCGAGTACACGTCGTCGTTTCGGAGGAGCAGGGTTGGCTTTTCGTAGGAATATACGA**T**GGTTTCAACGGTCCAGATGCGCCTGATTATCTCCTCTCTCATCTTTATCCTACGCTTCATCAGGAGCTCAAAGACTTGTTGTTATGGGACGATCCACGTGCTCAAAATAGTGATCATTGTGAGCGACGGTGGAGATGTGAGTGGGATCAAGAAAATCAAGATCCCCACCGTCGGTTAAAGGAACATATCGATCGTGGAAG |
| 9 | >B03_998898  CTTAGAGGTTCTTCTTCTTCTAAAGTTTACAAATAGAAACAAGAGGAAGAGCAGTAAAAGCATCAATGCCACTGATACGAACTTCCACAGCTTCCTATCATCCATTTCTCCAAAAACGTTTTGTAGATCAACAATGCTTCTGTTAGACTGCACACACAACACCAACATTGTGAATGAGAAAATTTGAGCAAGTCTTATTT**G**GATCAAGAAGAGGGACTTACTGTTGAAACCATCTTCTTTGAGACAAGTTCATGTTCCTTGACAAAGATTTGTTTTGCATACTTGTAAAGCTCCACATCAAGATTGTTAAGCGCTTTGATCTGCTGAATGACCTCTCTAGGAACTCTTGTACGCGTCTGTAAAATGCGTTATTGATTATTCGGGTCTTCTCTCATTTGACT |
| 10 | >B04_1922364  CTCACAATCTGTCACGCTTGTGGCATCAAAGCACCCGTGTTGGCAAAAAACATCTTGAAATGCACCCAAACCCACCTCCCCTTTTCTGCATTTTCAATTATCAAACATTTTAAAACTTAATCCATAACAGTACTCGATGTGTTTAAGATAAACATAATAGTAAAATGTACAAATATCTACTTTACCTTTGTGGATTTGAG**G**GAGAGAAGCCTAGGGGAAACCTTGTACAAGTCTTCATCCACAGGCTTAGGCGTCGCCGTTCTCCGCTTCACCACCGGTGTTGGACAGCTCCTCGGCGCGTTAGAATCACGCGCATCCTTCTTGAAGTTGTGTTGCTCCTTGGAGCTGGTTCTCTTTGGCTCCTGGCCCACCTTAGCCTATGAAGAAACAGTTACCATATA |
